# Portable Multispectral Imaging System for Sodium Nitrite Detection via Griess Reaction on Cellulose Fiber Sample Pads

**DOI:** 10.3390/s25237323

**Published:** 2025-12-02

**Authors:** Chanwit Kataphiniharn, Nawapong Unsuree, Suwatwong Janchaysang, Sumrerng Lumjeak, Tatpong Tulyananda, Thidarat Wangkham, Preeyanuch Srichola, Thanawat Nithiwutratthasakul, Nattaporn Chattham, Sorasak Phanphak

**Affiliations:** 1 Department of Industrial Physics and Medical Instrumentation, Faculty of Applied Science, King Mongkut’s University of Technology North Bangkok, Bangkok 10800, Thailand; s6004033920012@email.kmutnb.ac.th (C.K.); thidarat.w@sci.kmutnb.ac.th (T.W.); 2 Department of Physics, Academic Division, Chulachomklao Royal Military Academy, Nakhon Nayok 26001, Thailand; nawapong.un@crma.ac.th (N.U.); suwatwong.ja@crma.ac.th (S.J.); sumrerng.lu@crma.ac.th (S.L.); 3 Plant Biology & Astrobotany Laboratory, School of Bioinnovation and Bio-Based Product Intelligence, Faculty of Science, Mahidol University, Bangkok 10400, Thailand; tatpong.tul@mahidol.ac.th; 4 Kasetsart Agricultural and Agro-Industrial Product Improvement Institute, Kasetsart University, Chatuchak, Bangkok 10900, Thailand; aappua@ku.ac.th (P.S.); thanewat.n@ku.th (T.N.); 5 Cellulose for Future Materials and Technologies Special Research Unit, Department of Biotechnology, Faculty of Agro-Industry, Kasetsart University, Chatuchak, Bangkok 10900, Thailand; 6 Department of Physics, Faculty of Science, Kasetsart University, Bangkok 10900, Thailand; nattaporn.c@ku.th

**Keywords:** multispectral imaging (MSI), normalized difference index (NDI), computer vision, paper-based sensor, Griess reaction, sodium nitrite detection

## Abstract

This study presents a custom-built, portable multispectral imaging (MSI) system integrated with computer vision for sodium nitrite detection via the Griess reaction on paper-based substrates. The MSI system was used to investigate the absorption characteristics of sodium nitrite at concentrations from 0 to 10 ppm across nine spectral bands spanning 360–940 nm on para-aminobenzoic acid (PABA) and sulfanilamide (SA) substrates. Upon forming azo dyes with N-(1-naphthyl) ethylenediamine (NED), the PABA and SA substrates exhibited strong absorption near 545 nm and 540 nm, respectively, as measured by a spectrometer. This agrees with the 550 nm MSI images, in which higher sodium nitrite concentration regions appeared darker due to increased absorption. A concentration-correlation analysis was conducted for each spectral band. The normalized difference index (NDI), constructed from the most and least correlated bands at 550 nm and 940 nm, showed a stronger correlation with sodium nitrite concentration than the single best-performing band for both substrates. The NDI increased the coefficient of determination (R^2^) by approximately 19.32% for PABA–NED and 19.89% for SA–NED. This improvement was further confirmed under varying illumination conditions and through comparison with a conventional smartphone RGB imaging approach, in which the MSI-based NDI showed substantially superior performance. The enhancement is attributed to improved contrast, illumination normalization by the NDI, and the narrower spectral bands of the MSI compared with RGB imaging. In addition, the NDI framework enabled effective image segmentation, classification, and visualization, improving both interpretability and usability and providing a practical guideline for developing more robust models with larger training datasets. The proposed MSI system offers strong advantages in portability, sub-minute acquisition time, and operational simplicity, enabling rapid, on-site, and non-destructive chemical analysis.

## 1. Introduction

Detection of sodium nitrite (NaNO_2_) is important in medical, and particularly in food safety applications [1]. The substance is commonly used as a preservative for processed meat products such as bacon, ham, and sausage. It is also used to enhance the appetizing taste and smell, as well as change the color of processed meat to a pink-red and improve its texture [2,3,4,5]. However, sodium nitrite poses an adverse effect on health, since it is known to react with stomach acid and secondary amine metabolites, changing their structure to nitrosamines, which have been identified as a cause of stomach and esophageal cancer [2,6,7,8]. Moreover, discharges and wastewater treatment from the meat processing industry are the main sources of sodium nitrite contamination in surface and groundwater [9]. Therefore, standards have been established for the amount of sodium nitrite used. Global health regulators’ safety standards typically limit nitrite to no greater than 1 part per million (ppm) in drinking water [10,11] and 80 ppm in processed meat products for safe consumption [12].

Traditional techniques for measuring sodium nitrite include high-performance liquid chromatography (HPLC) [13,14,15], mass spectrometry (MS) [16,17], and gas chromatography (GC) [18]. However, these methods suffer from significant practical limitations that hinder their use for rapid, on-site screening. Their drawbacks include expensive costs of equipment and maintenance, long analysis time, as well as a requirement for complicated sample preparation and highly skilled workers. Furthermore, their lack of portability makes them unsuitable for field applications where immediate results are needed.

Colorimetric Griess reaction provides a simpler and faster method for quantitative analysis of nitrite. The chemical reaction unfolds in two steps. First, an aromatic amine, such as sulfanilamide (SA) [19,20] and para-aminobenzoic acid (PABA) [21], reacts with nitrite under acidic conditions to form a diazonium salt. Second, the diazonium salt binds to electron-rich coupling agent (e.g., N-(1naphthyl) ethylenediamine-NED), causing electron transitions. The result is the formation of a pink-to-magenta azo dye that exhibits high absorbance at its peak wavelength (approx. 545 nm). This measured absorbance is directly proportional to the initial sodium nitrite concentration [1,22,23]. Several development platforms of Griess reactions, such as Griess reagent-doped hydrogel sensor [23,24], nanoparticles [20,25,26], and paper-based platforms [27,28,29,30,31,32] are designed and examined in order to enable the rapid, visual detection of nitrite without the need for sophisticated procedures.

The paper-based platforms are interestingly effective, particularly for colorimetric methods involving the NED reagent, due to the inherent properties of the cellulose matrix. Cellulose is naturally hydrophilic and porous, which allows for the spontaneous transport of aqueous samples via capillary action, ensuring consistent interaction with the pre-deposited reagents. Furthermore, its inert nature and brilliant white background paper provides a perfect high-contrast stage for the Griess reaction’s colorimetric endpoint. This makes even subtle changes in the resulting azo dye’s pink-to-magenta hue easily distinguishable by the naked eye, turning a simple piece of paper into a sensitive analytical device [25,32]. Although visually interpreting the results by comparing the color with a standard strip is the simplest and most economical method, it has limitations in accuracy and depends on the observers. To solve this problem, digital cameras, such as those on smartphones, are now popularly used to measure color intensity, which provides more accurate results and reduces the gap between observation and laboratory measurement [22,23,29,33,34]. However, being limited to only three broad color channels, conventional RGB cameras lack the chemical specificity required to distinguish subtle spectral changes from background noise and are sensitive to light variation, which can compromise quantitative accuracy [35,36,37,38]. Spectroscopic approaches such as ultraviolet–visible spectroscopy (UV-VIS), Fourier Transform Infrared spectroscopy (FTIR), and Raman spectroscopy can be used to provide significantly better spectral information than digital camera [20,39,40,41]. While spectrometers provide more accurate quantitative data, they can only obtain an average measurement of measured regions on the sample and cannot provide spatial distribution information.

To overcome this limitation, either multispectral imaging (MSI) or hyperspectral imaging (HSI) systems can be employed. Unlike a spectrometer, MSI and HSI systems capture images, providing critical spatial distribution information that spectrometers inherently lack. Multispectral cameras (MSC) and hyperspectral cameras (HSC) provide more detailed spectral information than conventional RGB camera because they can record information in multiple narrow spectral channels, allowing them to capture finer spectral features and subtle changes in material properties that are not possible with traditional broad RGB channels [35,36,37,38,42]. Although HSI systems offer more spectral bands than MSI systems, they are typically more expensive, more complex to operate, slower in acquisition, and require more extensive data processing. In contrast, MSI systems, with carefully selected spectral bands, provide a cost-effective solution that is well suited for performing specific targeted tasks. A number of field applications employ the normalized difference index (NDI) approach, which relies on only a few highly informative spectral bands for data analysis [43,44]. The advantages of the NDI method include rapid image acquisition and straightforward interpretation. Therefore, the MSI system combined with NDI-based spectral analysis represents an effective approach for studying the absorption characteristics of sodium nitrite at different concentrations on a paper-based platform.

In this paper, the cellulose paper-based Griess reaction for sodium nitrite detection with the custom-built portable MSI system is investigated. To the best of our knowledge, this approach of integrating MSI with the Griess reaction for sodium nitrite analysis on paper platforms has not yet been reported. The MSI system was constructed using a Raspberry Pi 5 (Raspberry Pi Ltd, Cambridge, UK) as the core processor and a motorized filter wheel equipped with nine bandpass filters, each having a full width at half maximum (FWHM) ranging from 12 nm to 40 nm. This configuration allows light at specific wavelengths to pass through, providing more selective measurements compared to conventional RGB cameras. The results demonstrate that the developed MSI system effectively transforms conventional colorimetric detection into a quantitative imaging approach through the application of the NDI. The proposed NDI-based method significantly enhances the correlation with sodium nitrite concentration compared with single-wavelength analysis across different substrates and lighting conditions and also outperforms a conventional smartphone RGB imaging approach. In addition, the NDI framework enables effective image segmentation, classification, and visualization, thereby improving interpretability and supporting rapid, on-site, and non-destructive chemical analysis.

## 2. Materials and Methods

### 2.1. Sample Preparation for Standard Reagent Solutions

Sodium nitrite (NaNO_2_), sodium nitrate (NaNO_3_), sodium carbonate (Na_2_CO_3_), potassium nitrate (KNO_3_), hydrochloric acid (HCl), N-(1-naphthyl) ethylenediamine (NED), sulfanilamide (SA), para-aminobenzoic acid (PABA), and Cellulose fiber sample pads were purchased from Sigma-Aldrich (Singapore). All reagents and solvents obtained from commercial sources were of analytical grade. Deionized water (ELGA OPTION R-60, Celle, Germany) was used in all experiments. To obtain standard reagent solutions, stock solutions of sodium nitrite, sodium nitrate, sodium carbonate and potassium nitrate (100 ppm) were prepared by dissolving 10 mg of the substances in 100 mL of DI water. Then, a series of working standard solutions were obtained by diluting the original stock solutions to the required concentration (10 ppm) with DI water. SA (1 mM) was prepared by dissolving 17.2 mg of SA in 100 mL of DI water with strong stirring until dissolved. NED (2 mM) was prepared by dissolving 51.8 mg of NED in 100 mL of DI water. PABA (7 mM) was prepared by dissolving 4.8 mg of PABA and diluting it to a final volume of 5 mL with DI water, followed by 2 h of strong stirring at room temperature.

### 2.2. Characterization of SA-NED and PABA-NED Griess Reactions Using UV-VIS Spectrometer

To compare the Griess reactions of SA-NED and PABA-NED with nitrite ions, first, a series of standard sodium nitrite solutions (0, 0.5, 1, …, 10 ppm), described in Section 2.1, were mixed with SA solution in equal proportion by volume under acidic condition (HCl pH 1). After that, NED was added in ratio 2:1 *v*/*v* to obtain SA-NED Griess reaction. PABA-NED Griess reactions with sodium nitrite were also carried out under the same conditions. All solutions were characterized using LAMBDA 365 UV-VIS spectrometer (PerkinElmer, Shelton, CT, USA) to obtain standard spectral absorbance curves of the standard solutions at different sodium nitrite concentrations: 0, 0.5, 1, 2, 3, 4, 5, 6, 7, 8, 9, and 10 ppm (0–0.145 mM), respectively.

### 2.3. Specificity Test of PABA-NED Griess Reaction

To test selectivity of the PABA-NED reaction, same experimental procedure and conditions, as in Section 2.2, were carried out with 10 ppm solutions of potential interfering ions (sodium nitrate, sodium carbonate, and potassium nitrate). The resulting reactions were photographed for visual confirmation, and the absorption spectra were recorded to determine whether these interfering ions produced a significant false-positive signal compared to true response from sodium nitrite.

### 2.4. Custom-Built Multispectral Imaging System

For the subsequent parts of our experiment, a multispectral imaging (MSI) system was utilized. The MSI system was custom-built using a Raspberry Pi 5 as the core processor, a monochrome CMOS camera (HuaTeng Vision, Shenzhen, China Model No. HT-SUA134GM-T) for image acquisition, and two halogen lamps (Osram, Munich, Germany Model No. 64250 HLX). The MSI system has approximate dimensions of 19 cm × 24 cm × 30 cm and a total weight of 1.4 kg. The core of the imaging system is a filter wheel equipped with nine distinct bandpass filters, selected based on availability at the time of development. Each filter permits transmission of a specific wavelength range, enabling the system to acquire images across a broad spectral span from the ultraviolet (UV) to the near-infrared (NIR) regions. The specifications of each bandpass filter are provided in Table 1.

**Figure 1:**
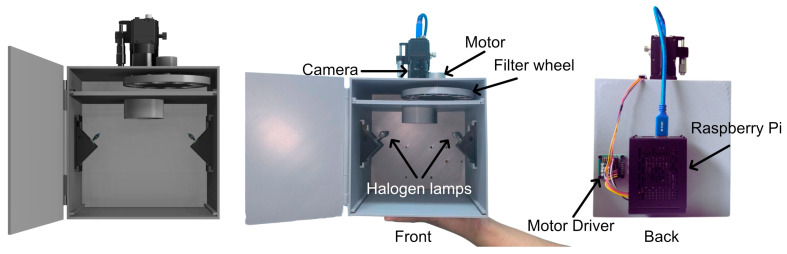
The custom-built multispectral camera (MSC) system. The figure displays (**left**) a 3D rendering of the device housing, (**middle**) a front view showing the internal imaging components including the camera, filter wheels, and halogen lamps, and (**right**) a rear view showing the electronic control unit composed of the Raspberry Pi and motor driver.

To create a portable and reproducible measurement tool, the housing and structural components of the MSC, shown in Figure 1, were fabricated using 3D printing. The figure illustrates the self-contained and integrated design of the system. The 3D rendering (left panel) displays the enclosed, box-like housing that prevents light from outside to enter. The front view (middle panel) reveals the precise arrangement of the core imaging components: the monochrome camera is mounted centrally at the top, positioned above the motorized filter wheel. The sample area below is illuminated by two angled halogen lamps, a configuration designed to provide uniform and consistent lighting for every measurement. The rear view (right panel) shows the neatly integrated electronic control unit, where the Raspberry Pi and the motor driver are mounted externally for easy access and heat dissipation. This fully integrated design, made possible by the flexibility of 3D printing, ensures that all components are held in a fixed geometry, which is crucial for achieving reproducible results.

### 2.5. Sample Preparation for Paper-Based PABA-NED Nitrite Detection Kit

**Figure 2:**
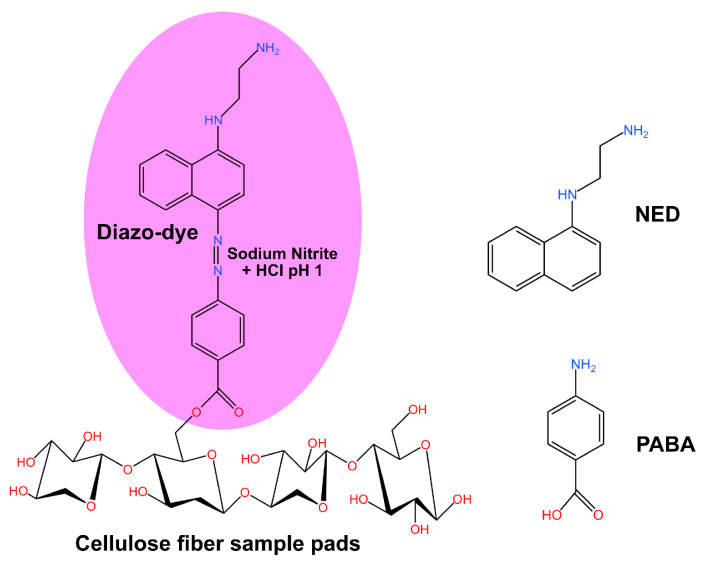
Chemical reaction schematic diagram of the Griess reaction for the determination of sodium nitrite, showing the formation of a diazo-dye from the reaction between PABA and NED in acidic conditions (HCl pH 1) on the cellulose fiber sample pads [21].

In this section we construct Griess-cellulose paper platform for nitrite detection. One advantage of using cellulose paper is that cellulose fibers possess a hydroxyl group can bind to a –COOH functional group at the end of PABA structure (Figure 2), allowing a resulting pink-to-magenta azo dye to be concentrated on the paper surface, hence enhancing the optical absorption signal [21]. To construct a detection kit, approximately 4 × 4 mm cellulose fiber paper pads were cut and immersed in the prepared 7 mM PABA solution. Then, they were left to dry at 37 °C for 2 h. The dried PABA-impregnated sample pads were then attached to a backing paper (Whatman No. 1 filter paper) to form a complete detection kit. Then, 1:1 sodium nitrite standard solution in HCl pH 1 were prepared at sodium nitrite concentrations of 0, 0.5, 1, 2, 3, 4, 5, 6, 7, 8, 9, and 10 ppm, respectively. Then a drop of each nitrite solution concentration was dropped onto each sample pad, followed by a drop of NED solution at the same volume ratio as nitrite. They were then left for 5 min before either RGB or multispectral images would be taken.

### 2.6. Image Acquisition and Data Analysis Methods

Each spectral band image was normalized using both the white and dark references. For each band, the white reference was captured using the same white cellulose filter paper on which the samples were placed, while the dark reference was acquired with the illumination turned off and the lens aperture fully closed to represent the camera’s dark current. Each band was then normalized using Equation (1):

(1) $$I_{\mathrm{norm}\;}=\;\frac{I\;-\;I_{\mathrm{dark}}}{I_{\mathrm{white}}\;-\;I_{\mathrm{dark}}}$$

where $I_{\mathrm{norm}}$ is the normalized pixel intensity of the sample for a given band, I is the corresponding pixel intensity of the sample, $I_{\mathrm{white}}$ is the corresponding pixel intensity of the white reference, and $I_{\mathrm{dark}}$ is the corresponding pixel intensity of the dark reference.

For every sample pad with known nitrite concentration provided in a ground-truth image, the average image intensity of the corresponding sample pad was extracted for each spectral band. A correlation analysis was then performed to quantify the relationship between the normalized image intensity in each spectral band and the known sodium nitrite concentration. From this analysis, the spectral band exhibiting the strongest correlation (the most sensitive band) and the band with the weakest correlation (the least sensitive band) were identified. The least sensitive band effectively serves as a baseline, helping to correct for variations in illumination. To create a bounded normalized metric ranging from −1 to 1, a Normalized Difference Index (NDI) was calculated [35]. This index utilizes the best and worst correlating bands to enhance image contrast. The NDI for each pixel was calculated using the following Equation (2):

(2) $$\mathrm{NDI}\;=\;\frac{I_{\mathrm{best}}\;-\;I_{\mathrm{worst}}}{I_{\mathrm{best}}\;+\;I_{\mathrm{worst}}}$$

where $I_{\mathrm{best}\;}$ is the intensity of the pixel in the best correlating band, and $I_{\mathrm{worst}}$ is the intensity of the same pixel in the worst correlating band. The resulting NDI image provides a powerful representation of the nitrite concentration, normalized against potential experimental variations. To validate this approach, the relationship between the mean NDI value of each sample pad and its known concentration was evaluated using statistical methods, such as calculating the coefficient of determination (R^2^) and Pearson correlation coefficient (*r*).

### 2.7. Smartphone-Based RGB Imaging and Comparative Validation

To validate the efficacy of the MSI-based NDI, a comparative analysis was performed against a conventional mobile phone RGB imaging approach using a commercially available smartphone (Xiaomi Redmi Note 13 Pro, Xiaomi Corporation, Beijing, China). For data processing, the RGB images were decomposed into individual red, green, and blue channels using a Python 3.13.5 script executed in Visual Studio Code 1.104.2 (Universal). In addition to the individual channels, the Normalized Grayscale Intensity (NGI) was also calculated using Equation (3) [45]:

NGI = 255 − (0.2126 × R + 0.7152 × G + 0.0722 × B)(3)

where R, G, and B correspond to the pixel intensities of the red, green, and blue channels, respectively. The NGI and all individual RGB channels were then subjected to correlation analysis to assess their relationships with sodium nitrite concentration and to compare their performance against the MSI-based NDI approach.

### 2.8. Image Segmentation and Classification Strategy

Finally, computer vision techniques were employed for image segmentation and classification to isolate the region of interest and subsequently categorize sodium nitrite concentrations based on mean NDI values. In the preprocessing stage, a median filter with a 5 × 5 kernel was applied to the 550 nm and 940 nm images to suppress salt-and-pepper noise while preserving edge integrity. This was achieved by replacing each pixel intensity with the median of its 25-pixel (5 × 5) neighborhood. Following noise reduction, segmentation was performed using a predefined NDI threshold to automatically remove the background and exclude samples corresponding to 0 ppm sodium nitrite. Morphological operations were then applied to refine the segmentation mask and eliminate small, isolated artifacts. Mean NDI values were calculated for the segmented regions and used to classify the samples into four concentration levels: low, medium, high, and very high. The performance of the segmentation and classification procedures was assessed using the Intersection over Union (IoU) metric computed against the ground truth using Equation (4) [46]:

(4) $$\mathrm{IoU}\;=\;\frac{\left|A\cap B\right|}{\left|A\cup B\right|}$$

where A∩B represents the overlapping area between the predicted classification and the ground truth regions, and A∪B represents their combined area.

## 3. Results and Discussion

### 3.1. Comparison of PABA-NED and SA-NED Spectral Characteristics

**Figure 3:**
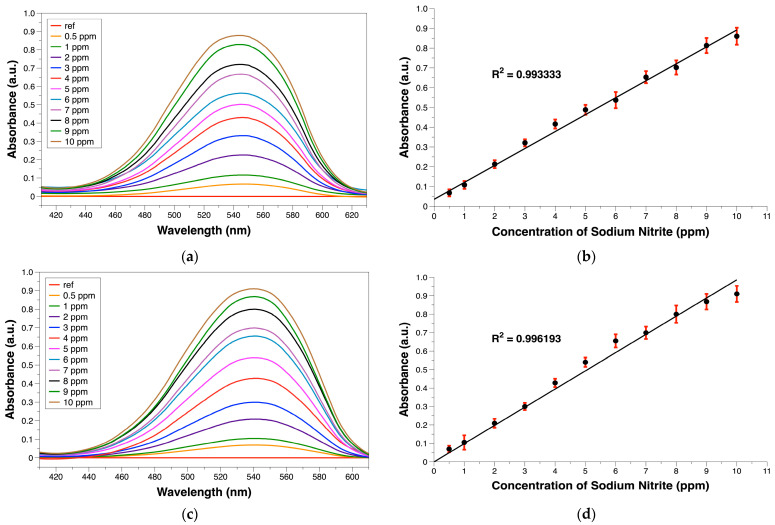
The comparison of sodium nitrite measurement results using PABA-NED and SA-NED: (**a**) The UV-VIS absorbance spectrum of standard solution using PABA-NED; (**b**) Standard curve of PABA-NED using maximum absorbance at 545 nm (R^2^ = 0.993); (**c**) The UV-VIS absorbance spectrum of standard solution using SA-NED; (**d**) Standard curve of SA-NED using maximum absorbance at 540 nm (R^2^ = 0.996).

In this section, we compared the sodium nitrite UV-VIS absorption curves of SA-NED and PABA-NED, as described in Section 2.2. Figure 3a,c show the absorption spectra of PABA-NED and SA-NED, respectively, with the sample solutions at sodium nitrite concentrations ranging from 0.5 to 10 ppm. The results showed that both methods yielded consistent outcomes: as the sodium nitrite concentration increased, the absorbance also increased. Then, the maximum absorbance values at 540–545 nm obtained from the spectra were used to create calibration curves Figure 3b,d, to analyze the linear relationship between concentration and absorbance. It was found that both methods gave excellent linearity in the tested concentration range. Both SA-NED and PABA-NED methods yielded a coefficient of determination (R^2^) of 0.99, confirming that the measurements have high accuracy and can be reliably used for quantitative analysis. The method also proved highly selective on nitrite detection.

### 3.2. Selectivity of the PABA-NED Griess Reaction

**Figure 4:**
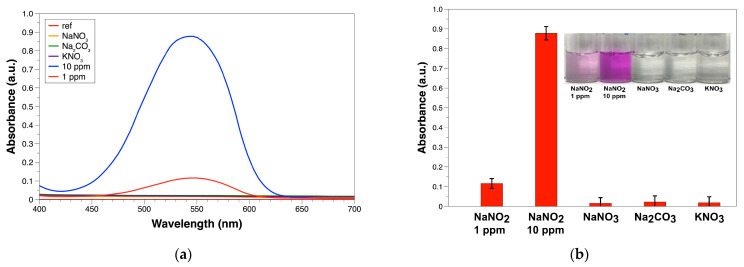
Specificity test results of the PABA-NED method: (**a**) UV-VIS absorption spectra comparing sodium nitrite (1 and 10 ppm) and sodium nitrate, sodium carbonate, potassium nitrate at a concentration of 10 ppm; (**b**) Bar chart comparing the maximum absorbance at 545 nm and the color photograph of the actual solution (inset) after the reaction.

This section shows the results of the specificity test with nitrite ion from the UV-VIS absorption spectra. The absorption spectra in Figure 4a clearly shows that the sodium nitrite at concentration of 1 and 10 ppm exhibit a clear absorption peak at a wavelength of approximately 545 nm. The other three interfering ion solutions, even at concentrations as high as 10 ppm, did not show any significant absorbance signal, with the curves overlapping close to the reference line.

From the inset in Figure 4b, the bar chart and solution photograph further confirm this result. It can be seen that the absorbance of sodium nitrite at 1 and 10 ppm is significantly higher than that of the other interfering ions. This is consistent with the color changes observed with the naked eye, with only sodium nitrite producing pink (1 ppm) and magenta (10 ppm) colors, while the other interfering ion solutions remained colorless. This experimental result confirms that the developed PABA-NED detection method has high selectivity for nitrite ion detection. There are no significant false-positive signals from common interfering ions, e.g., nitrate; only nitrite is specific for this detection method. The double-bond of nitrogen is required to create the magenta AZO dye, where nitrate cannot directly form the bond via the Griess process.

### 3.3. Absorption Study with the MSI System

**Figure 5:**
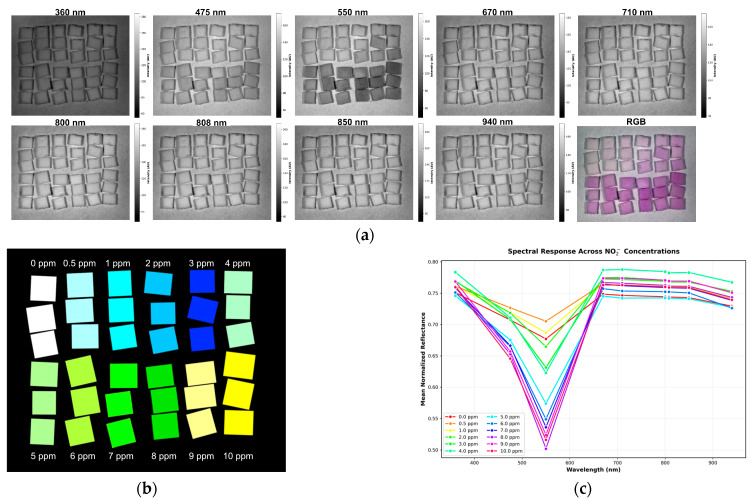
(**a**) The raw multispectral images (nine spectral bands) and the combined RGB image (constructed from the 475 nm, 550 nm, and 670 nm bands); (**b**) Ground-truth image defining the regions of interest (ROIs) with known sodium nitrite concentrations; (**c**) The spectral curves showing the mean intensity of each concentration region plotted against the different spectral bands (wavelengths).

The samples were placed inside the multispectral imaging (MSI) system and captured using a Python script executed in Visual Studio Code. The MSI system sequentially acquired one image per spectral band across nine central wavelengths including 360 nm, 475 nm, 550 nm, 670 nm, 710 nm, 800 nm, 808 nm, 850 nm, and 940 nm, with the entire acquisition completed under one minute. The raw multispectral images, along with the combined RGB image constructed from the 475 nm, 550 nm, and 670 nm bands, are presented in Figure 5a. The regions of interest in each spectral band image were spatially mapped to corresponding regions with known sodium nitrite concentrations, as defined in the ground-truth image shown in Figure 5b. The mean intensity of each concentration region was then calculated for every spectral band and plotted as spectral curves representing different concentrations, as illustrated in Figure 5c. The results from Figure 5 enable the analysis of the absorption characteristics of sodium nitrite on a paper-based platform (in this case, PABA-NED) through the reflectance intensities captured by the MSI system. For instance, in the 550 nm image, regions with higher sodium nitrite concentrations appear darker (indicating higher absorption) than those with lower concentrations. Similarly, the 475 nm image exhibits a trend but with lighter intensity variations. In contrast, minimal intensity variation is observed across the remaining spectral bands. These findings indicate that sodium nitrite on the PABA-NED substrate exhibits its strongest absorption at 550 nm, followed by 475 nm, while showing minimal absorption at 360 nm, 670 nm, 710 nm, 800 nm, 808 nm, 850 nm, and 940 nm. This behavior is further confirmed by the spectral curves in Figure 5c, where the reflectance at 550 nm decreases markedly (indicating higher absorption) with increasing sodium nitrite concentration—consistent with the spectroscopy measurements presented in Figure 3a.

**Figure 6:**
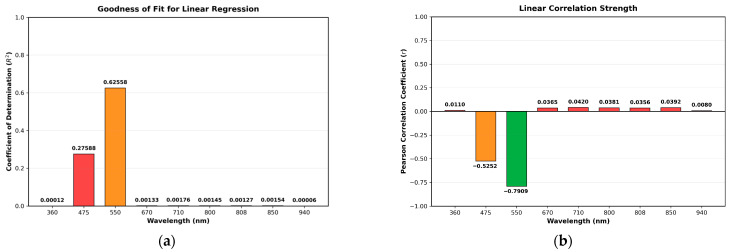
Correlation analysis summary for the nine spectral bands: (**a**) Coefficient of Determination (R^2^) and (**b**) Pearson Correlation Coefficient (*r*).

To determine which spectral bands, exhibit the strongest and weakest correlation with sodium nitrite concentration, a correlation analysis was performed. For each band, the mean intensity of every concentration region was plotted against the corresponding known concentrations, and the relationship was modeled using linear regression. From these fits, the coefficient of determination (R^2^) and the Pearson correlation coefficient (*r*) were extracted to quantify the strength of the correlation between each spectral band and sodium nitrite concentration. As shown in Figure 6, the 550 nm band demonstrated the strongest correlation, with R^2^ and |r| values of 0.62558 and 0.7909, respectively. In contrast, the 940 nm band exhibited the weakest correlation, with R^2^ and |r| values of 0.00006 and 0.0080, respectively. It is worth noting that the correlations of most other bands—except for the 475 nm band—are relatively similar. Therefore, any band with a correlation level comparable to that of the weakest-correlated band may also be used in constructing the NDI map.

**Figure 7:**
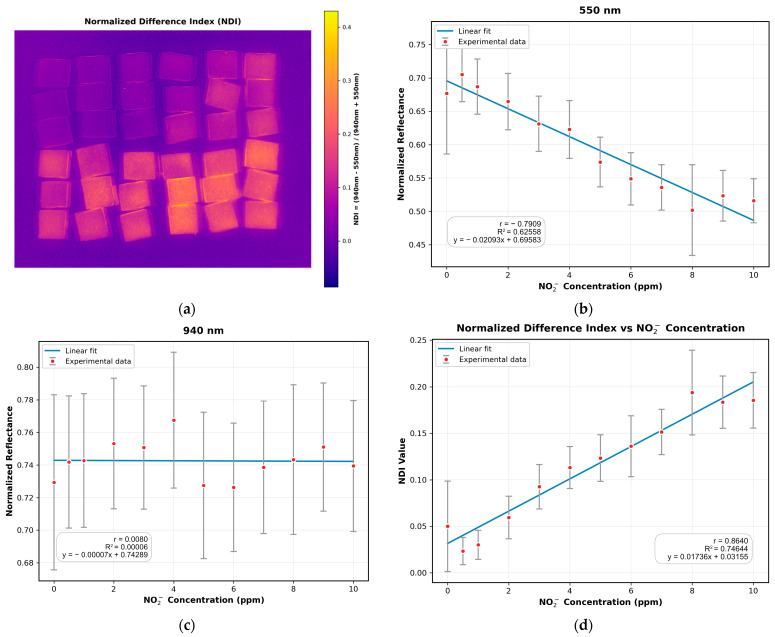
Analysis and validation of the Normalized Difference Index (NDI): (**a**) The computed NDI image, derived from the 550 nm and 940 nm bands; (**b**) Linear regression fitting for the strongly correlated 550 nm band; (**c**) Linear regression fitting for the weakly correlated 940 nm band; (**d**) Linear regression plot for the NDI, demonstrating an enhanced correlation with sodium nitrite concentration compared to the best single band.

The NDI image, shown in Figure 7a, was computed using the spectral band images with the highest and lowest correlations identified previously, and the results of the fitting are shown in Figure 7b,c, respectively. The correlation analysis for the NDI image was conducted following the same procedure as before. As presented in Figure 7d, the NDI exhibited an enhanced correlation with sodium nitrite concentration compared to the best single-correlated band (550 nm), achieving R^2^ and |r| values of 0.74644 and 0.8640, respectively. The improvements of 19.32% in R^2^ and 9.24% in |r| are attributed to the NDI’s increased robustness against illumination variations and its improved contrast, resulting from the combination of the strongly correlated and weakly correlated spectral band images.

**Figure 8:**
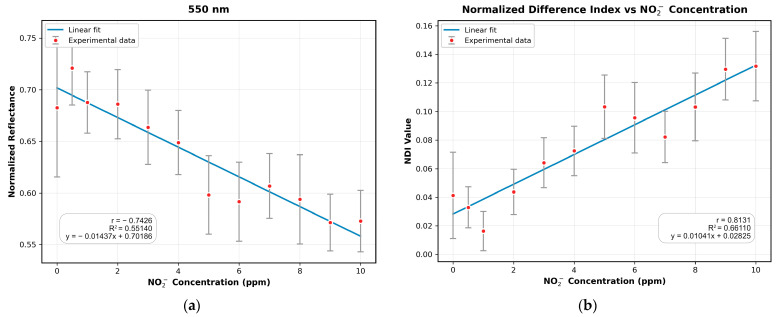
Validation of the correlation analysis using the SA-NED substrate: (**a**) Linear regression fitting for the best single-correlated band (550 nm); (**b**) Linear regression fitting for the NDI (computed using the 550 nm and 940 nm bands), showing an enhanced correlation.

To validate these findings at different substrates, the same correlation analysis was performed using the SA-NED substrate. The results exhibited a consistent trend, with the strongest absorption occurring at 550 nm, followed by 475 nm, and minimal absorption observed at 360 nm, 670 nm, 710 nm, 800 nm, 808 nm, 850 nm, and 940 nm. Moreover, the correlation between the NDI image (computed using the 550 nm and 940 nm images) and sodium nitrite concentration showed improvements of 19.89% and 9.49% in R^2^ and |r|, respectively, compared to the 550 nm image. The fitted results for the 550 nm and NDI images are presented in Figure 8a,b, respectively.

**Figure 9:**
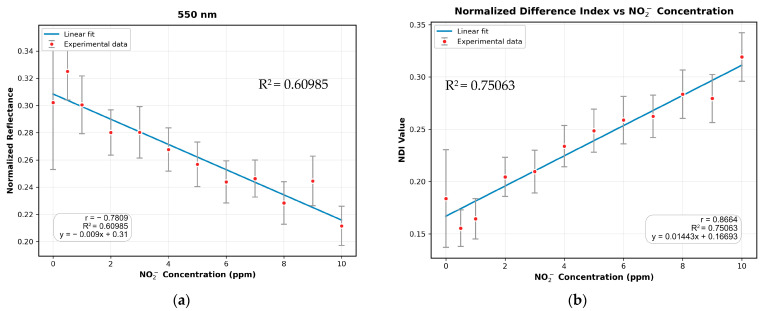

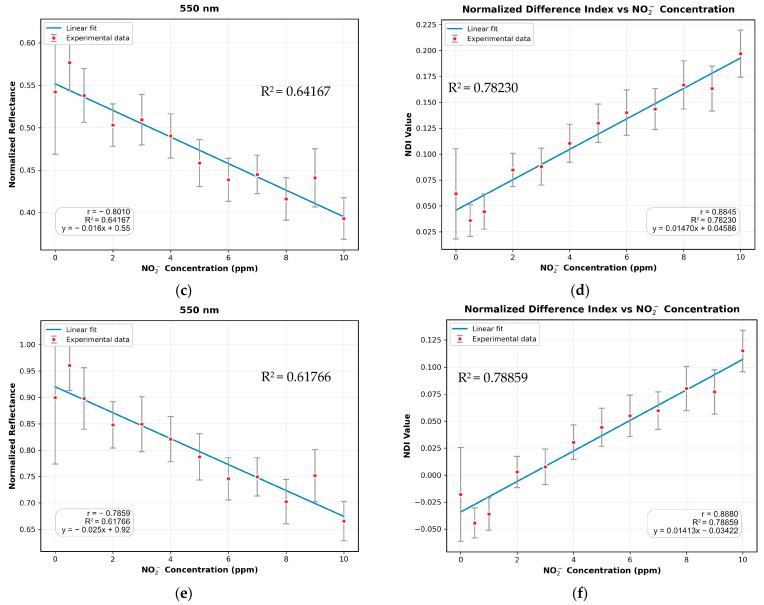
Validation of the correlation analysis across different lighting conditions by varying illumination voltages (3.5 V, 4.0 V, and 4.5 V): (**a**,**c**,**e**) Linear regression fitting for the best single-correlated band (550 nm) at 3.5 V, 4.0 V, and 4.5 V, respectively; (**b**,**d**,**f**) Linear regression fitting for the NDI (computed using the 550 nm and 940 nm bands) at 3.5 V, 4.0 V, and 4.5 V, respectively.

In addition, a comparative correlation analysis under different illumination conditions was performed to verify the enhancement of the NDI–sodium nitrite concentration correlation. The brightness of the halogen lamps was adjusted by setting their input voltages to 3.5 V, 4.0 V, and 4.5 V. The corresponding linear regression fits and R^2^ values for each condition are presented in Figure 9a–f. Across all lighting levels, the NDI approach (computed using the 550 nm and 940 nm bands) consistently outperformed the best single-band method (550 nm), yielding an average improvement of 24.22% in R^2^.

### 3.4. Comparison of MSI-Based NDI and Mobile Phone RGB Imaging for Sodium Nitrite Detection

**Figure 10:**
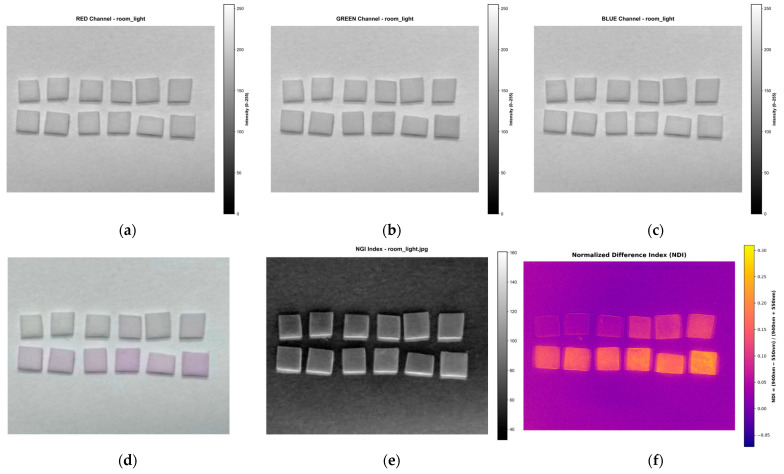
Visual comparison of smartphone RGB imaging and the MSI: (**a**–**c**) Decomposed smartphone channels showing (**a**) red, (**b**) green, and (**c**) blue; (**d**) original RGB image; (**e**) NGI image computed from the smartphone RGB data; (**f**) NDI image generated using the MSI system (computed using the 550 nm and 940 nm bands).

To validate the efficacy of the MSI-based NDI, a comparative analysis was conducted against a traditional mobile phone RGB imaging approach. A Xiaomi Redmi Note 13 Pro was used to capture RGB images of sodium nitrite deposited on a PABA-NED substrate under standard room lighting conditions. Figure 10a–c show the extracted red, green, and blue channels, respectively, from the RGB image presented in Figure 10d. The Normalized Grayscale Intensity (NGI) map, computed using Equation (3), is shown in Figure 10e for comparison with the NDI map generated by the MSI system (Figure 10f).

**Figure 11:**
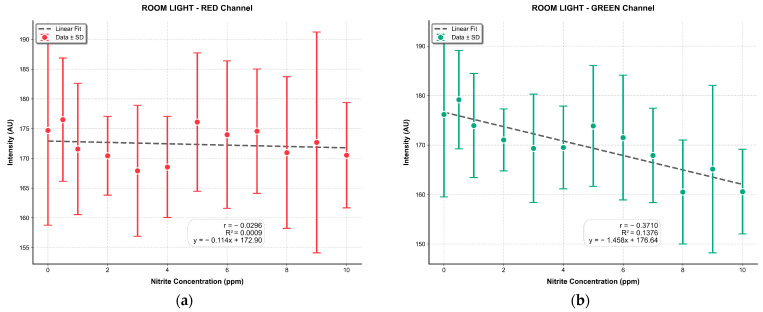

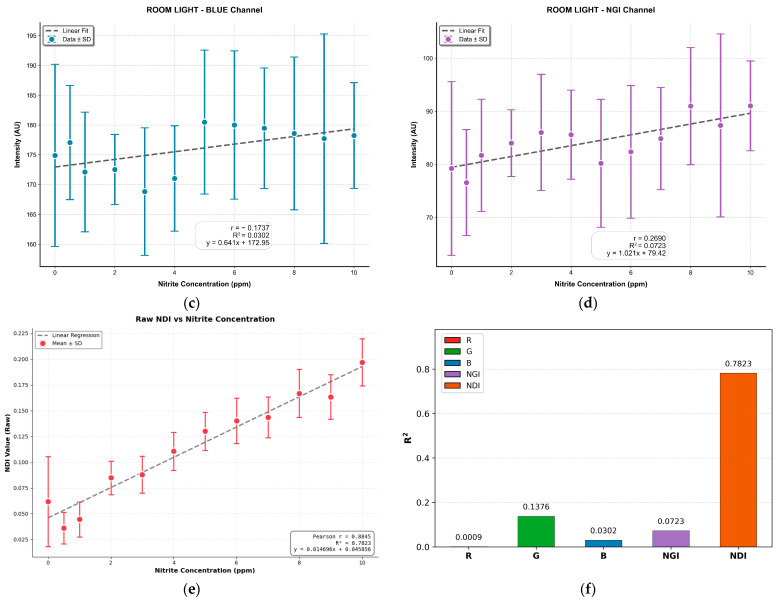
Correlation analysis of sodium nitrite concentration on the PABA-NED substrate for: (**a**) red channel, (**b**) green channel, and (**c**) blue channel of the smartphone RGB image; (**d**) NGI image computed from the smartphone RGB data; (**e**) NDI image generated using the MSI system (using the 550 nm and 940 nm bands); and (**f**) summary bar chart comparing the R^2^ values, demonstrating the superior performance of the MSI-based NDI over all smartphone-based approaches.

Correlation analyses for the red, green, and blue channels, as well as the NGI and NDI, were performed in the same manner as previously described. The results, presented in Figure 11, reveal that the RGB channels and the NGI map exhibit weak correlations with sodium nitrite concentration, yielding low R^2^ values of 0.0009, 0.1376, 0.0302, and 0.0723, respectively, with the green channel performing the best among them. In contrast, the NDI approach demonstrated a substantially stronger correlation, achieving an R^2^ value of 0.7823. A comparison of these R^2^ values, shown in Figure 11f, highlights the markedly enhanced correlation achieved by the NDI relative to the mobile phone RGB imaging approach.

### 3.5. Segmentation and Classification

**Figure 12:**
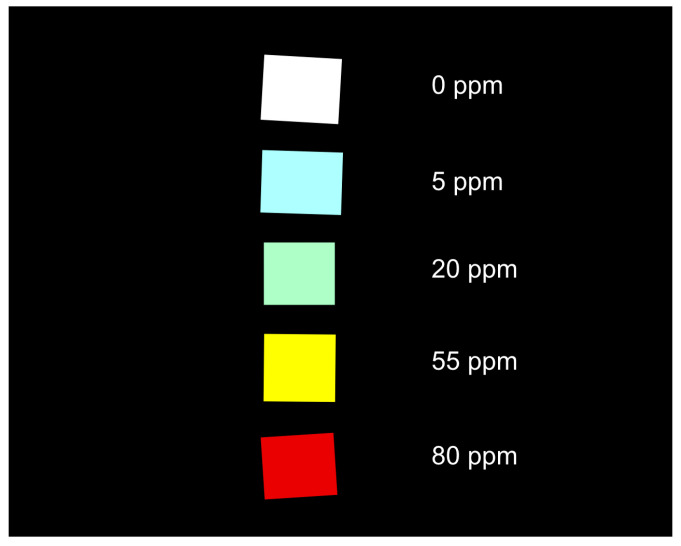
The ground-truth image showing the arrangement of the five sodium nitrite samples used in the processing pipeline, with concentrations of 0, 5, 20, 55, and 80 ppm arranged from top to bottom.

This section provides a practical guideline for developing a classification of sodium nitrite concentrations. In this example, five samples with sodium nitrite concentrations of 0, 5, 20, 55, and 80 ppm were arranged from top to bottom on the ground-truth image, as shown in Figure 12.

**Figure 13:**
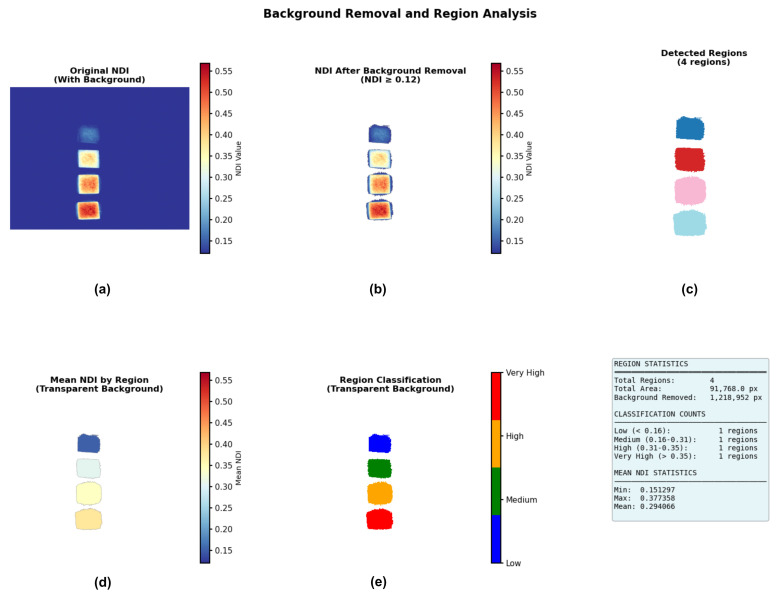
The NDI-based image processing and classification pipeline: (**a**) The calculated NDI image, computed from the 550 nm and 940 nm bands after noise reduction; (**b**) Segmentation result after applying an NDI threshold (0.12) to remove the background and 0 ppm sample; (**c**) The four segmented sample regions after morphological cleaning; (**d**) A plot of the mean NDI value calculated for each of the four segmented regions; (**e**) The final classification map showing the four concentration levels (Low, Medium, High, Very High) based on defined NDI thresholds.

**Figure 14:**
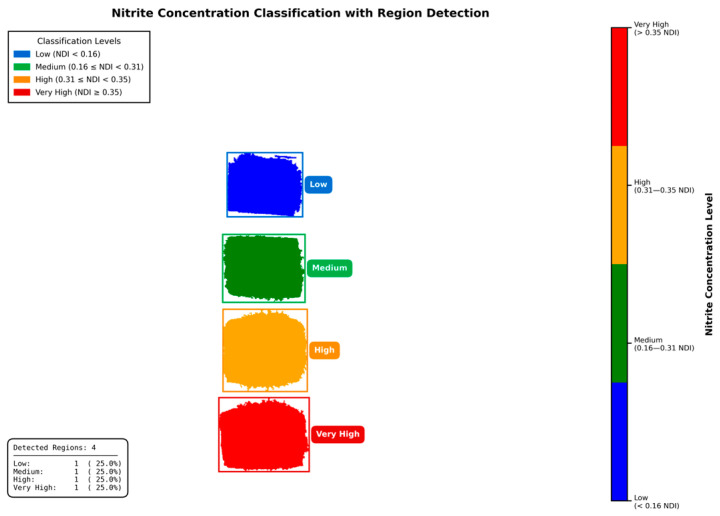
The final visualization of the classification results showing object detection boxes with their corresponding class labels overlaid on the image for interpretation.

First, noise reduction was applied to both the best-correlating band (550 nm) and the weakest-correlating band (940 nm) using a median filter to remove salt-and-pepper noise while preserving edge features. The NDI image in Figure 13a was then calculated using these two spectral band images. By applying a specific NDI threshold (0.12 in this case), the background and the 0 ppm sample were segmented out, leaving only the regions containing sodium nitrite, as shown in Figure 13b. Morphological cleaning was subsequently performed to remove small isolated regions, improving the segmentation of relevant samples. At this stage, four samples with different sodium nitrite concentrations were clearly segmented, as shown in Figure 13c. The mean NDI value for each segmented region was then calculated and plotted in Figure 13d. Based on these mean NDI values, classification was performed using defined NDI thresholds to categorize four concentration levels: low (0.12 ≤ NDI < 0.16), medium (0.16 ≤ NDI < 0.31), high (0.31 ≤ NDI < 0.35), and very high (NDI ≥ 0.35), as shown in Figure 13e. Finally, object detection boxes with class labels were overlaid for improved visualization and interpretation, as shown in Figure 14.

**Figure 15:**
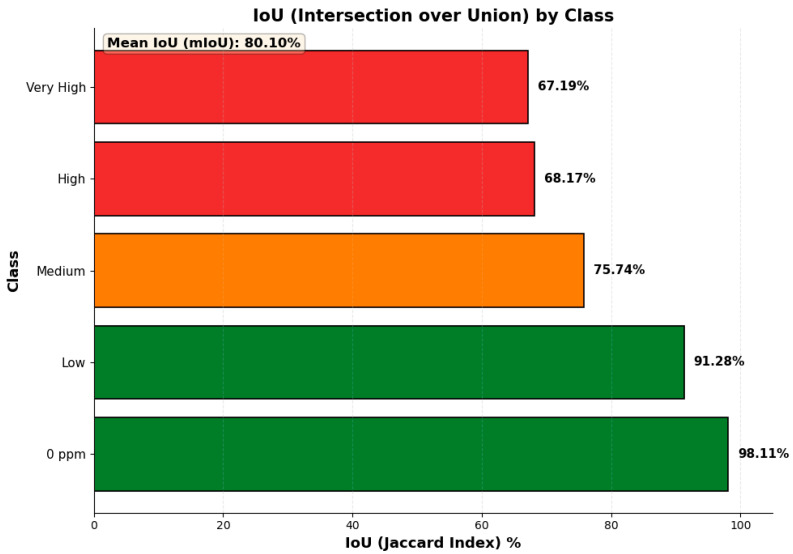
Intersection over Union (IoU) scores for each class, demonstrating a mean IoU (mIoU) of 80.10%.

To evaluate the performance of the segmentation and classification, a pixel-level analysis was performed by comparing the predicted classification map with the ground truth. The results, summarized in Figure 15, present the Intersection over Union (IoU) metric calculated using Equation (4). The system achieved a mean IoU (mIoU) of 80.10%, with IoU scores of 98.11%, 91.28%, 75.74%, 68.17%, and 67.19% for the Background (0 ppm), Low, Medium, High, and Very High classes, respectively.

This example demonstrates the effectiveness of using NDI-based imaging with defined thresholds combined with computer vision techniques to enhance the visualization and interpretation of sodium nitrite concentrations. At this stage, the threshold values for each class were manually selected to align with the ground truth, serving as an initial guideline for classification. In our subsequent work, we plan to develop a more robust approach by collecting additional data to determine these thresholds statistically or by employing machine-learning methods in combination with various NDI maps to achieve more accurate and reliable classification.

## 4. Conclusions

This study demonstrates the application of a custom-built, portable multispectral imaging (MSI) system integrated with computer vision for the quantitative analysis of sodium nitrite on a paper-based platform using the Griess reaction. By employing the normalized difference index (NDI), conventional colorimetric detection was effectively transformed into a robust quantitative imaging approach, yielding enhanced correlation with sodium nitrite concentration for both PABA and SA substrates and under varying illumination conditions. Compared with single-wavelength analysis, the NDI achieved stronger analytical performance due to its improved image contrast and robustness against illumination variation. The spectral behavior observed from the MSI system closely agreed with UV–VIS spectroscopic measurements, confirming the accuracy and reliability of the proposed method while additionally providing spatial information unavailable from conventional spectroscopy. Furthermore, the MSI-based NDI significantly outperformed a conventional smartphone RGB imaging approach, attributable to the narrower spectral bandwidths of the MSI system. The NDI framework also enabled effective image segmentation, classification, and visualization, improving both interpretability and usability. With its portability, sub-minute acquisition time, and operational simplicity, the developed MSI system is well suited for rapid, on-site, and non-destructive chemical analysis. Future work will focus on developing more robust nitrite concentration estimation models by integrating NDI features with advanced machine learning techniques to further enhance the system’s analytical and diagnostic capabilities.

## Figures and Tables

**Table 1:** Specifications of the bandpass filters used in the MSI system.

Bandpass Filters	CWL	FWHM	Model/Part No.	Manufacturers
UV	360 nm	~40 nm	BP360	HuaTeng Vision, Shenzhen, China
Blue	475 nm	~18 nm	BP475	Nano Macro Optics Co., Ltd., Shenzhen, China
Green	550 nm	~22 nm	BP550	Nano Macro Optics Co., Ltd., Shenzhen, China
Red	670 nm	~26 nm	BP670	Nano Macro Optics Co., Ltd., Shenzhen, China
Red Edge	710 nm	~12 nm	BP710	Nano Macro Optics Co., Ltd., Shenzhen, China
NIR	800 nm	~30 nm	BP800	Nano Macro Optics Co., Ltd., Shenzhen, China
NIR	808 nm	~40 nm	BP808	HuaTeng Vision, Shenzhen, China
NIR	850 nm	~40 nm	FS03-BP850	HuaTeng Vision, Shenzhen, China
NIR	940 nm	~40 nm	FS03-BP940	HuaTeng Vision, Shenzhen, China

## Data Availability

The original contributions presented in the study are included in the article, further inquiries can be directed to the corresponding author.
